# Determinants of hospital length of stay for patients with schizophrenia

**DOI:** 10.1192/j.eurpsy.2024.1557

**Published:** 2024-08-27

**Authors:** M. Turki, M. Barkallah, N. Boussaid, A. labyadh, S. Ellouze, N. Halouani, J. Aloulou

**Affiliations:** Psychiatrie B, CHU Hedi Chaker, Sfax, Tunisia

## Abstract

**Introduction:**

relapse and frequent rehospitalizations. The length of stay (LOS) of these patients has been a concern of researchers. The ability to identify determinants of LOS at admission – and, thus, identify patients who are likely to need a longer stay early on – may help treatment planning.

**Objectives:**

We aimed to investigate socio-demographic and clinical profile of inpatients with schizophrenia, and to identify factors associated with LOS.

**Methods:**

It was a retrospective study carried out among 90 inpatients with schizophrenia admitted to the psychiatry “B” department, Hedi Chaker university hospital (Sfax, Tunisia), during the period between January 2015 and December 2019. Data collection was performed through the patients’ medical records. Statistical analysis was performed using SPSS v.25.

**Results:**

The mean age of our patients was 32 years. Among them, 57.78% were women. The mean LOS was 28 days. Factors found to be significantly associated with LOS were: the number of admissions (p<0.001, r=0.404), involuntary hospitalization (p=0.001), violence and disturbance of public order as a reason of admission (p < 0001) and the lack of social support (p=0.039). As for the clinical symptoms, hallucinations were significantly associated with a longer LOS (p=0.001).

**Conclusions:**

Our findings highlighted several factors associated with a longer LOS. This may be helpful to the management of hospitalization and ensuring that any periods of liberty deprivation do not last longer than necessary to provide appropriate treatment.

**Disclosure of Interest:**

None Declared

